# Radiogenomics and Texture Analysis to Detect von Hippel–Lindau (VHL) Mutation in Clear Cell Renal Cell Carcinoma

**DOI:** 10.3390/cimb46040203

**Published:** 2024-04-08

**Authors:** Federico Greco, Valerio D’Andrea, Bruno Beomonte Zobel, Carlo Augusto Mallio

**Affiliations:** 1 Department of Radiology, Cittadella Della Salute Azienda Sanitaria Locale di Lecce, Piazza Filippo Bottazzi 2, 73100 Lecce, Italy; 2 Research Unit of Radiology, Department of Medicine and Surgery, Università Campus Bio-Medico di Roma, Via Alvaro del Portillo 21, 00128 Roma, Italy; valerio.dandrea@unicampus.it (V.D.); b.zobel@policlinicocampus.it (B.B.Z.); c.mallio@policlinicocampus.it (C.A.M.); 3 Fondazione Policlinico Universitario Campus Bio-Medico, Via Alvaro del Portillo 200, 00128 Roma, Italy

**Keywords:** clear cell renal cell carcinoma, kidney cancer, radiogenomics, radiomics, texture analysis, *VHL*

## Abstract

Radiogenomics, a burgeoning field in biomedical research, explores the correlation between imaging features and genomic data, aiming to link macroscopic manifestations with molecular characteristics. In this review, we examine existing radiogenomics literature in clear cell renal cell carcinoma (ccRCC), the predominant renal cancer, and von Hippel–Lindau (*VHL*) gene mutation, the most frequent genetic mutation in ccRCC. A thorough examination of the literature was conducted through searches on the PubMed, Medline, Cochrane Library, Google Scholar, and Web of Science databases. Inclusion criteria encompassed articles published in English between 2014 and 2022, resulting in 10 articles meeting the criteria out of 39 initially retrieved articles. Most of these studies applied computed tomography (CT) images obtained from open source and institutional databases. This literature review investigates the role of radiogenomics, with and without texture analysis, in predicting *VHL* gene mutation in ccRCC patients. Radiogenomics leverages imaging modalities such as CT and magnetic resonance imaging (MRI), to analyze macroscopic features and establish connections with molecular elements, providing insights into tumor heterogeneity and biological behavior. The investigations explored diverse mutations, with a specific focus on *VHL* mutation, and applied CT imaging features for radiogenomic analysis. Moreover, radiomics and machine learning techniques were employed to predict *VHL* gene mutations based on CT features, demonstrating promising results. Additional studies delved into the relationship between *VHL* mutation and body composition, revealing significant associations with adipose tissue distribution. The review concludes by highlighting the potential role of radiogenomics in guiding targeted and selective therapies.

## 1. Introduction

Renal cell carcinoma (RCC) is growing in incidence and stands as the most prevalent form of kidney cancer. According to epidemiological statistics, RCC constitutes the majority (90%) of kidney cancer cases, predominantly comprising clear cell RCC (ccRCC) at 70%, papillary RCC at 10–15%, and chromophobe RCC at 5%. The remaining subtypes are less frequent (≤1%) [1].

In recent years, the remarkable advancements of new genetic sequencing techniques and the accessibility of genomic information through open source data from the Human Genome Project have transformed biomedical research, together with the introduction of a new field known as radiogenomics [2,3]. Radiogenomics delineates the correlation between the imaging characteristics of a specific pathology, referred to as the imaging phenotype, and the genomics of the diseases (such as gene expression patterns, gene mutations, and other genome-related features). The objective is to establish links between macroscopic features and molecular elements, aligning these imaging features with biological behavior and outcomes [4,5].

Imaging phenotypes indeed represent the macroscopic expression of processes occurring at the molecular level that is detectable with imaging [i.e., computed tomography (CT) and magnetic resonance imaging (MRI)] [5]. Combining the former and the latter is important to understand tumor biology and to analyze the intrinsic tumor heterogeneity and fingerprints, with implications for patient care. One of the great advantages of radiogenomics is that it allows one to obtain data that are not always available with genomic tests on biopsy specimens, since both the gene expression and gene mutations are analyzed on small samples and not on the entire tumor lesion, possibly not capturing the whole heterogeneity of the disease which is typical in ccRCC [6].

Radiologists can obtain imaging features either qualitatively (i.e., measuring and describing phenotypical characteristics) or by quantitative variables (i.e., measures, degree of contrast uptake/washout). Indeed, a more complex connection between individual image pixel cannot be observed by the naked eye. Converting the above relationships into minable quantitative data is the practice of radiomics, a growing field of radiological research, where the tumor phenotype is used for applications ranging from improved diagnostics to prognostication to therapeutic response prediction [7,8].

Radiogenomics also proved to be an ideal solution for predicting gene mutations in ccRCC. With the improvements in diagnostic tests like CT and MRI imaging, the incidence of patients diagnosed with RCC has surged in recent decades and the most frequent type of RCC, based on histological and molecular subtypes, is clear cell carcinoma, frequently associated with mutations in the von Hippel–Lindau (*VHL*) gene [9].

The aim of this literature review is to investigate the role of radiogenomics and texture analysis for detection *VHL* gene mutation in ccRCC patients, evaluated through retrospective analyses.

## 2. VHL Role in Clear Cell Renal Cell Carcinoma

The most observed gene mutation in ccRCC is the *VHL* tumor suppressor gene [2]. RCC genome sequencing has identified several mutations with prognostic meaning and connection with imaging features [4]. Somatic *VHL* mutations are identified in 30–60% of ccRCCs, accounting for the vast majority of sporadic ccRCCs [9]. CcRCC is also one of the most known cancers in terms of genetics and molecular mechanisms. It is characterized by the loss of the short arm of chromosome 3 (3p) which harbors the *VHL* tumor suppressor gene, whose function is to regulate the hypoxia inducible factor (HIF) protein. This protein also regulates the transcription of several genes, including vascular endothelial growth factor (VEGF), which regulate angiogenesis, proliferation, tumor cell migration, and permeability. *VHL* gene mutation can lead to an increase in HIF concentrations with the relative upregulation of the neoangiogenic cascade. The inactivation of the *VHL* genes interrupts the cellular degradation of HIF even in the presence of aerobic status, which leads to the pathogenesis of ccRCC. The overproduction of VEGF resulting in angiogenesis justifies the hypervascular feature of the ccRCC upon imaging [10,11].

VHL disease is a systemic condition with an autosomal dominant transmission, and the development of RCC is the result of *VHL* gene inactivation. The loss of 3p does not impact on the tumor size, grade, and metastatic spread, whereas mutations in gain of 5q are related to better prognosis [12,13]. The VHL complex is widely inactivated (over 80%) in ccRCC, by either mutation or methylation.

According to the previous statistics, the prevalence of *VHL* mutation is also confirmed by The Cancer Genome Atlas (TCGA) cohort, where the main percentage (52.3%) of ccRCC-mutated genes is represented by the *VHL* mutation group [14].

Over 800 *VHL* mutations have been identified in both hereditary and sporadic ccRCC [15]. More than 50% of these mutations are frameshift and nonsense mutations characterized by a high probability of causing a loss of VHL protein (pVHL) activity [16,17]. Since there are numerous missense mutations distributed across the three exons of *VHL*, the consequences of these alterations on the integrity of pVHL are difficult to predict. In relation to this, Reichsteiner et al. found three different groups of missense mutations: a first group that determines the severe destabilization of the pVHL, a second group that does not determine the destabilizing effects on the pVHL but is relevant for the interaction with HIFα, elongin B, and elongin C, and a third group with pVHL activity comparable to the wild type [18].

The presence of ccRCC in type 1 VHL disease is associated with nonsense and frameshift mutations generating *VHL*-null alleles, while type 2 VHL disease is mainly associated with missense mutations. This last type of mutation is further divided into type 2A with a low risk of developing ccRCC, type 2B with a high risk of developing ccRCC, and type 2C which predisposes to pheochromocytoma [19].

Regarding sporadic ccRCC, some groups found a correlation between “loss-of-function” mutations (nonsense and frameshift) and worse prognosis for patients or higher HIF target-directed response rates, while other groups did not confirm these findings [20].

## 3. Material and Methods

We conducted a quantitative synthesis encompassing various studies that examined different mutations in ccRCC, with a specific focus on studies involving *VHL* mutation. 

A comprehensive literature review by searching through multiple databases, including PubMed, Medline, Cochrane Library, Google Scholar, and Web of Science was performed. Our aim was to identify all relevant articles on the topic of radiogenomics and ccRCC. The search terms included “radiogenomics and …” one of the following MeSH search terms: “renal cell carcinoma”, “clear cell renal cell carcinoma”, “VHL”, ”radiomics”, “kidney cancer”, or “renal cancer”. Titles and abstracts of the articles retrieved from the above search were then selected for relevance. Inclusion criteria were publication in the English language, publication without limit of time span, and article topic pertaining to radiogenomics of ccRCC. Exclusion criteria encompassed publications not available in the English language, studies unrelated to ccRCC or radiogenomics, duplicate articles, and non-primary literature, such as abstracts and letters to the editor. The latter were excluded following a thorough review to ensure that no primary studies were overlooked [21].

To meet the study’s eligibility criteria, articles had to be published in a peer-reviewed journal. The screening process involved assessing the importance of articles based on their title and abstract. Articles lacking an abstract were excluded, and if there was uncertainty about inclusion or exclusion criteria based on the abstract, the full-text article was downloaded. To minimize selection bias and errors, two investigators (F.G. and V.D.) independently conducted the literature search for each publication and subsequently analyzed the extracted data.

All the studies included in our literature review are summarized in Table 1.

## 4. Discussion

The adoption of radiogenomics in the oncological field is promising and encouraging, especially for renal cancer and its most common type, namely ccRCC. The possibility of characterizing a renal lesion using CT/MRI imaging in relation to genetic, epigenetic, and pathologic heterogeneity without using invasive treatments definitively represents an advantage [31,32].

Moreover, in recent years, the implementation of the biomedical research into the genomic field is permitted by access to the open source data of the Human Genome Project. Many studies included in this review were based on TCGA, which is an atlas of genetic changes in the different types of cancer, including RCC. All the data, from medical images to tissue sampling from TGCA, relate to a single identifier and have free-access download [33,34].

Karlo et al. [22] investigated the connection between imaging features and gene mutations. Their study showed a great prevalence in his study population of *VHL* mutation (53.2%) and significant CT results related to the loss of this gene. Well-defined margins represented an important characteristic of ccRCC with a loss of *VHL* function (*p* = 0.013) and constituted a marker of less infiltrative behavior and lower aggressiveness compared to ccRCC with ill-defined margins. Another significant finding linked to *VHL* mutation was the nodular tumor enhancement (*p* = 0.021) of ccRCC and evidence of intratumoral vascularity (*p* = 0.018) at CT images, that could probably be explained by the fact that a loss of *VHL* function relates to the upregulation of hypoxia inducible factors and an overexpression of angiogenic factors. Moreover, the *VHL* gene mutation was significantly more common in solid than in cystic tumors (*p* = 0.016) [22].

Shinagare et al. [23] investigated similar imaging features to the previous studies, always analyzing the most frequent mutations. Moreover, in this study, *VHL* represented the predominant group of mutations (50.5%) but significant results were founded only for *BAP1* mutation and its association with ill-defined tumor margins and the presence of calcification and for *MUC4* mutation and its connection with exophitic growth. No imaging features were associated with *VHL* and the other mutations. In contrast to Karlo et al. [22], they did not find an association between the well-defined tumor margin and solid ccRCC with *VHL* mutation. This fact could be explained by the different imaging features analyzed and evaluated, for example, Shinagare et al. [23] contemplated a “well-defined” margin only when more than 90% of the entire tumor circumference was ‘pencil-thin’ sharp. They estimated the presence of necrosis only in tumors considered to be solid and did not consider low-attenuation cystic regions as necrotic. For this reason, the presence of intratumoral necrosis was definitively lower than it was in Karlo’s study. Lastly, the nodular enhancement, intratumoral vascularity, and renal vein invasion were not evaluated by Shinagare et al. [23] and all the data were screened by multiple readers from multiple institutions, whereas the study by Karlo et al. [22] was derived from a single institution.

Cen et al. performed a radiogenomic analysis of a tumor suppressor *RUNX3* (runt-related transcription factor 3) in ccRCC [35]. Radiogenomic analysis showed the presence of intratumoral vascularity in ccRCC with a high methylation level of the *RUNX3* gene, a feature also found in ccRCC with the *VHL* gene mutation, as found by Karlo et al. [22,35]. Furthermore, high *RUNX3* methylation levels presented an ill-defined tumor margin and left-sided tumors, two characteristics, respectively, not present in *VHL* gene mutation [22,35].

TCGA Research Network7 classified ccRCC into four categories (m1, m2, m3, and m4) based on the mRNA expression with different clinical and biological characteristics [36]. These four tumor categories showed different clinical and biological characteristics [37]. The analysis of Bowen et al. showed well-defined margin as the only radiogenomic feature of m1-subtype [37].

**Figure 1:**
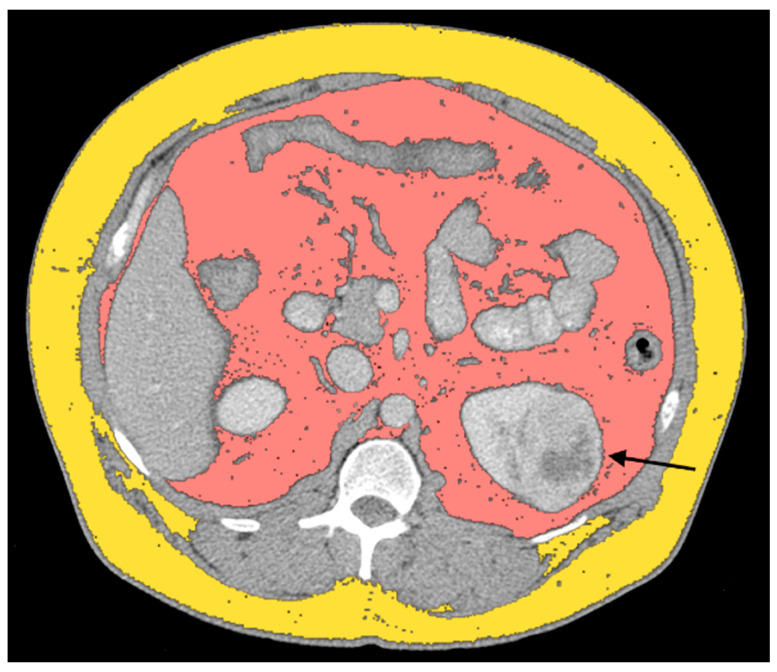
Axial CT image showing ccRCC with *VHL* mutation (black arrow), SAT, and VAT, segmented, respectively, in dark yellow and salmon pink.

Greco et al. explored the connection between *VHL* mutations and abdominal adipose tissue distribution of patients with ccRCC [24]. Interestingly, they found a significant increase in the total adipose tissue (TAT) and visceral adipose tissue (VAT) in the ccRCC-*VHL* group (Figure 1). The VAT has a key role in RCC pathogenesis, especially in obese patients. The VAT of pathologically obese patients exhibits high levels of HIF-1α [38]. The growth of adipose tissue determines the release of HIF-1, which is related to angiogenesis, tumor metastases, unfavorable prognosis, and tumor resistance to therapy. The pVHL in association with other proteins is responsible for the degradation of HIF; with *VHL* inactivation, the loss of HIF degradation leads to angiogenesis and cell growth. The increased VAT in ccRCC could be explained by the inactivity of the *VHL* gene that cannot modulate HIF due to its lack of degradation activity without pVHL. This theory may reveal the explanation of the significant increase in abdominal adipose tissue, in the ccRCC-*VHL* group [24].

Further confirmations about the association between the CT features of body composition and *VHL* mutation derived from another study by the same group [30]. In this retrospective study, they quantified TAT, VAT, subcutaneous adipose tissue, and the total abdominal muscle in ccRCC patients with *VHL* and *TTN* gene mutations. Their results showed a significant decrease in TAT, VAT, SAT, and TAM in the ccRCC-*TTN* group compared to the ccRCC-*VHL* group, establishing a better survival rate for the ccRCC-*VHL* group than the ccRCC-*TTN* one [30].

Future studies will analyze additional CT features not evaluated in ccRCC patients with *VHL* mutations (i.e., presence or absence of peritumoral collateral vessels) to obtain further insights into the phenotypic imaging of this gene mutation.

An important limitation could be represented by the retrospective investigation of all the studies mentioned above. The great chance of accessing the wide free public databases of imaging and genome data could implement a standardized model for the management of radiogenomics. This is especially the case if future research could be conducted to offer prospectively developed evidence and increase the size of the studied cohorts [31].

Li et al. performed radiomics analysis for *VHL* gene mutation prediction in ccRCC [25]. For each segmented 3D tumor, quantitative textures were extracted using an in-house developed program with features from each phase. The high-order spatial distributions of the intensity within the 3D tumor are described by texture features. Gray-level co-occurrence matrix (GLCM), gray-level run length matrix (GLRLM), gray-level size zone matrix (GLSZM), and neighborhood gray-tone difference matrix (NGTDM) methods were used to extract texture features from each phase. Finally, 156 features were extracted from each tumor from triphasic CT. The intraclass correlation coefficient (ICC) was used to measure the intra- and inter-observer repeatability. Features with an ICC of less than 0.85 were discarded. The correlation between imaging features and *VHL* mutation was evaluated using the Wilcoxon rank-sum test. Features selected by Boruta, a random forest (RF)-based wrapper algorithm [39], and by the minimum redundancy maximum relevance ensemble (mRMRe), a method designed to find a subset of both relevant and complementary features, were analyzed [40]. Benjamini–Hochberg method was used for multiple hypothesis correction using the false discovery rate (FDR) [41]. A statistically significant correlation was found between all eight mRMRe features and the *VHL* mutation (FDR-adjusted *p* < 0.05). Of the eight Boruta features, seven showed a statistically significant correlation with the *VHL* mutation before FDR correction (*p* < 0.05), while after FDR correction, five Boruta features showed a significant association (FDR-adjusted *p* < 0.05) [25].

Chen et al. proposed a new multi-classifier multi-objective (MCMO) radiogenomics predictive model [9]. During training, similarity-based sensitivity and specificity were defined and considered as the two objective functions simultaneously, to obtain more reliable prediction results. Several classifiers were used, and to take advantage of them, the evidential reasoning (ER) approach was used to fuse the output of each classifier. Furthermore, quantitative CT features were used by MCMO, trained by a new similarity-based multi-objective optimization algorithm (SMO) to predict *VHL* mutation. With this proposed MCMO model, a predictive area under the receiver operating characteristic curve (AUC) over 0.85 for *VHL* mutation with balanced sensitivity and specificity was obtained. Furthermore, MCMO produced more reliable results than other optimization algorithms and commonly used fusion strategies [9].

Lin et al. identified the radiomic subtypes of ccRCC patients [26]. Tumor segmentation was performed manually by a single radiologist using the ITK-SNAP software (version 3.8) [42]. The region of interest (ROI) was segmented on the largest tumor slice on the axial plane. To determine the tumor margins, postcontrast images were used during segmentation. Intelligence Foundry (GE Healthcare, version 1.3) was used for radiomics feature extraction. In total, 122 radiomic features were extracted: 18 first-order features, 23 gray-level co-occurrence matrix features, 16 gray-level run-length matrix features, 16 gray-level size-zone matrix features, 5 neighboring gray-tone difference matrix features, 13 gray-level dependence matrix features, 18 shape features, and 13 textural phenotype features [26]. The Image Biomarker Standardization Initiative standard was fully met by all 122 radiomic features. The R package “CancerSubtypes” was used to perform consensus clustering to identify the intrinsic radiomic subtypes of ccRCC patients, according to this unsupervised method [43]. The three-cluster solution, based on the consensus clustering of the radiomic features, was identified as the optimal solution: the consensus cumulative distribution function and delta plot showed a minimum relative change between k = 3 and k = 4, the consensus matrix heatmap showed three clusters with significant interconnectivity, and the mean silhouette distance for k = 3 (0.81) was greater than that for k = 2 (0.71) or k = 4 (0.75) but did not present significant negative values. Consequently, ccRCC patients were divided into three subtypes: cluster 1 (c1), cluster 2 (C2), and cluster 3 (C3). *VHL* had a high mutation rate in all subtypes. *VHL* gene mutation was observed less frequently in the C1 subtype than in the C2 and C3 subtypes (*p* = 0.038) [26].

Zeng et al. evaluated the feasibility of predicting the molecular characteristics by CT in ccRCC [27]. Four algorithms for feature selection (GBDT, LASSO, RF, XGBoost), and eight algorithms as classifiers (RF, GBDT, AdaBoost, LR, DT, SVM, NB, KNN) were used. Among the 32 algorithm combinations, RF showed the best performance on the test set. The radiomics models based on RF were able to predict *VHL* gene mutation (AUC = 0.971) [27].

Currently, a significant portion of radiogenomics investigations has centered on CT imaging, likely attributed to its status as the primary imaging modality for assessing renal cancer globally.

Zhi-Cheng Li et al. [28] introduced a comprehensible radiomics model by the extracting pertinent features from multiphasic CT scans to distinguish between ccRCC and non-ccRCC. Fifty-two texture features were derived from each phase using various methods, such as the gray-level co-occurrence matrix (GLCM), gray-level run length matrix (GLRLM), gray-level size zone matrix (GLSZM), and neighborhood gray-tone difference matrix (NGTDM). Ultimately, 156 features were extracted from the triphasic CT for each tumor, using a random-forest-based wrapped algorithm (Boruta) and minimum redundancy maximum relevance enable (mRMRe). The biological significance of radiomics was explored by examining the potential radiogenomics connection between the imaging characteristics and a critical ccRCC driver gene—the *VHL* mutation. The model, comprising eight relevant features, achieved an AUC of 0.949 and an accuracy of 92.9%. Five features were notably linked to the *VHL* mutation (FDR *p* < 0.05). The radiogenomics findings demonstrated that all eight mRMRe features were notably linked to the *VHL* mutation. Nevertheless, following FDR correction, three of the Boruta features (f5, f6, f7) did not show a significant association with the *VHL* mutation. This suggests that, while all-relevant features were identified, they may not all be relevant to *VHL* mutation, and imaging features specifically related to *VHL* may be less effective in distinguishing ccRCC from non-ccRCC [28].

MRI is arousing interest, with the total absence of radiations and the availability of obtaining further information from a great variety of sequences, such as T2-weighted and diffusion weighted imaging, which may develop image prediction models by providing additional radiogenomic features [29].

In our review, all the studies mentioned earlier focused on CT-imaging features. Only the study of Shinagare et al. partially explored the MRI features, considering a limited portion of their investigation, specifically analyzing MRI features in 22 patients out of the total group of 103 [23].

The only study exclusively relying on MRI was conducted by Anari et al. [29]. They developed an MRI-based algorithm to predict the volumetric growth rate category of tumors.

They selected patients with an ccRCC and *VHL* mutation that underwent MRI at least at two different points. It was demonstrated that that machine learning employing radiomic features can be used for accurately predicting the tumor growth rate. However, no imaging characteristics were associated with *VHL* mutations. Considering the benefits derived from the absence of exposure to radiological risk and the availability of multiple sequences at our disposal, a greater emphasis on magnetic resonance studies should be considered in the future.

## 5. Target Therapies and Future Strategies for VHL ccRCC

We have seen how, in today’s context, radiogenomics contributes to the characterization of an oncological lesion without resorting to invasive treatments. Similarly, the increasingly targeted and selective therapies should be considered to achieve greater efficacy and reduced toxicity.

Intratumoral hypoxia is linked to the advancement of tumors and resistance to therapy. The *VHL* tumor suppressor gene was discovered in 1993, and subsequent investigations unveiled that the gene’s product, pVHL, collaborates with other proteins to construct the VBC complex [44,45]. The VBC complex operates as an E3 ubiquitin ligase and oversees the levels of the α-subunit of the HIF transcription factor. HIF oversees numerous genes necessary for cellular adaptation and survival in hypoxic conditions, underscoring pVHL’s pivotal role in oxygen-sensing pathways. Individuals affected by VHL disease, possessing a germline mutation of the *VHL* gene, manifest renal cell carcinomas and a series of tumors displaying hypervascular characteristics. Comprehensive research elucidating *VHL*’s function has contributed to pioneering first-in-human drugs, such as belzutifan, an HIF-2α inhibitor [46].

When contemplating therapeutic approaches directed at HIF in clear cell renal cell carcinoma (ccRCC) cells, it is imperative to ascertain whether HIF serves as the primary driver for the tumor.

It has been demonstrated that HIF-1α hinders the tumor growth of RCC by suppressing cell-cycle progression regulated by c-Myc [47]. Taken together, the findings from Takamori’s et al. research and those of other groups distinctly suggest that HIF-2 could potentially act as a driver in RCC [46,48]. Considering these findings, efforts were undertaken to develop inhibitors specifically targeting HIF-2. Among the HIF-2α inhibitors, belzutifan stands out as the most advanced compound in clinical development, with a phase II trial conducted in VHL patients in both the United States and Europe with 61 patients enrolled [47].

Most of these patients (56 out of 61, or 91.8%) exhibited a decrease in the size of RCC tumors following the treatment, with 30 cases (49%) achieving a reduction in tumor size (partial response, PR) exceeding 30%. In light of these findings, in 2021, the FDA granted approval for belzutifan for adult patients with VHL disease in need of therapy for RCC, central nervous system hemangioblastoma, or pancreatic neuroendocrine tumors when immediate surgery is not deemed necessary [46,47].

Moreover, anthiangiogenic therapies (sunitinib, sorefanib, axitinib, or bevacizumab) have demonstrated an increased response in ccRCC patients with “loss-of-function” mutations (nonsense, frameshift, and in frame), blocking several of the downstream effects of pVHL [49]. Further studies will perform radiogenomic and texture analyses to detect specific patterns related to the type of *VHL* mutation in ccRCC.

Radiogenomics and texture analysis are able to evaluate the molecular basis from the acquired imaging data, allowing the detection of *VHL* mutation in ccRCC. The loss of *VHL* function with the consequent accumulation of HIF proteins [50,51] results in the uncontrolled activation of HIF-targeted genes that regulate angiogenesis, glycolytic metabolism, and apoptosis [52]. Therefore, ccRCCs are rich in glycogens, lipids, and are highly vascularized [52,53], which could underline the radiological features visible in contrast-enhanced CT images. To validate this hypothesis, high-throughput radiomics data were used to determine which radiologic characteristics are subtype-discriminative and biologically important. This can be achieved by selecting all-relevant features, so as to select a target-relevant feature subset that provides meaningful insights into the investigated target itself [54].

## 6. Conclusions

In the era of precision medicine, radiogenomics potentially plays a predominant role in predicting the tumor response to treatment by integrating the imaging observations with genomic data and assessing the composition of the mass instead of solely relying on tissue sampling, which may underestimate the dominant molecular pattern due to intra-tumoral heterogeneity.

While most studies focused on CT imaging, emphasizing its role as the primary modality for renal cancer assessment, it is likely that a growing interest in exploring MR studies will be observed. Future investigations should consider prospective approaches and expand the analysis of imaging features associated with *VHL* mutation, providing a more comprehensive understanding of the radiogenomic landscape in ccRCC. These findings could enable the acquisition of data on the *VHL* expression via CT/MR imaging approaches and could be applied for the development of targeted therapy, aiming to achieve the imaging signatures of tumor genomics that can aid in identifying patients who benefit from specific targeted therapies.

## Figures and Tables

**Table 1:** Summary of radiogenomics and texture analysis studies.

Author/Year	Title	Number of Patients	Imaging Technique	Gene Studied	Outcomes Related with VHL Mutation	With/Without Texture Analysis
Karlo et al., 2014 [22]	Radiogenomics of Clear Cell Renal Cell Carcinoma: Associations between CT Imaging Features And Mutations	233	CT	*VHL*, *BAP1*, *KD5MC*	Mutations of *VHL* were significantly associated with well-defined tumor margins (*p* = 0.013), nodular tumor enhancement (*p* = 0.021), and gross appearance of intratumoral vascularity (*p* = 0.018).	Without
Shinagare et al., 2015 [23]	Radiogenomics of clear cell renal cell carcinoma: Preliminary findings of The Cancer Genome Atlas–renal cell carcinoma (TCGA-RCC) imaging research group	103	CT/MR	*VHL*, *BAP1*, *PBRM1*, *SETD2*, *KDM5C*, and *MUC4*	No significant results connected with *VHL* mutation. *BAP1* mutation was associated with ill-defined tumor margins and the presence of calcification (*p* = 0.02 and 0.002, respectively, Pearson’s χ^2^ test); *MUC4* mutation was associated with exophytic growth (*p* = 0.002, Mann–Whitney *U* test).	Without
Greco et al., 2021 [24]	Relationship between VAT and genetic mutations (*VHL* and *KDM5C*) in clear cell renal cell carcinoma	97	CT	*VHL*, *KD5MC*	Increased amount of TAT, especially VAT, in the ccRCC-*VHL* and ccRCC-*KDM5C* groups. Statistically significant differences (i.e., increase in the ccRCC-*VHL* group) between control group and ccRCC-*VHL* group were obtained for TAT area (*p* < 0.001) and VAT area (*p* < 0.01). Statistically significant differences (i.e., increase in the ccRCC-*KDM5C* group) between control group and ccRCC-*KDM5C* group were obtained for TAT area (*p* < 0.0001), VAT area (*p* < 0.0001), and SAT area (*p* < 0.01). The ccRCC-*KDM5C* group showed a statistically significant increase in TAT area (*p* < 0.05), VAT area (*p* < 0.05), and VAT/SAT ratio (*p* < 0.05) with respect to the ccRCC-*VHL* group.	Without
Li et al., 2018 [25]	Differentiation of clear cell and non-clear cell renal cell carcinomas by all-relevant radiomics features from multiphase CT: a *VHL* mutation perspective	85	CT	*VHL*	All-relevant features in corticomedullary phase CT can be used to differentiate ccRCC from non-ccRCC with high accuracy. Most RCC-subtype discriminative CT features were associated with the key RCC driven gene—the *VHL* gene mutation. Five out of eight all-relevant features were significantly associated with *VHL* mutation (false discovery rate adjusted *p* < 0.05).	With
Chen et al., 2018 [9]	Reliable gene mutation prediction in clear cell renal cell carcinoma through MCMO radiogenomics model	57	CT	*VHL*, *PBRM1*, *BAP1*	They proposed an MCMO radiogenomics predictive model. Using the proposed MCMO model, they achieved a predictive area under the receiver operating characteristic AUC over 0.85 for *VHL*, *PBRM1*, and *BAP1* genes with balanced sensitivity and specificity.	With
Lin et al., 2021 [26]	Radiomic profiling of clear cell renal cell carcinoma reveals subtypes with distinct prognoses and molecular pathways	160	CT	*VHL*, *MUC16*, *FBN2*, and *FLG* cell cycle related Pathways	Radiomic profiling revealed three ccRCC subtypes with distinct clinicopathological features and prognoses. *VHL*, *MUC16*, *FBN2*, and *FLG* were found to have different mutation frequencies in these radiomic subtypes. *VHL* had a high mutation rate in all the subtypes, and *VHL* mutations were less frequently observed in the C1 subtype than in the C2 and C3 subtypes. Based on TCGA-KIRC cohort, the K-M analysis also revealed that *VHL*, *FLG*, and *MUC16* mutation status did not show survival differences, while patients with *FBN2* mutation have a superior OS. The prognostic value of the radiomic subtypes was further validated in another independent cohort (log-rank *p* = 0.015).	With
Zeng et al., 2021 [27]	Integrative radiogenomics analysis for predicting molecular features and survival in clear cell renal cell carcinoma	207	CT	*VHL*, *BAP1*, *PBRM1*, *SETD2*, molecular subtypes (m1–m4)	They evaluated the potential value of CT radiomics features in classifying mutations and molecular subtypes of ccRCC, with the use of multiple machine learning algorithms. The radiomics models based on RF were able to predict *VHL* gene mutation (AUC = 0.971). High-risk group of validation set predicted by multi-omics model showed significantly poorer OS (HR = 6.20, 95%CI: 3.19–8.44, *p* < 0.0001).	With
Zhi-Cheng Li et al., 2019 [28]	Towards an interpretable radiomics model for classifying renal cell carcinomas subtypes: a radiogenomic assessment	255(188 cc RCC/67 non-ccRCC)	CT	*VHL*	All eight mRMRe features were significantly associated with *VHL* mutation (FDR-adjusted *p* < 0.05). After FDR correction, 3 all-relevant Boruta features (f5, f6, f7) were not significantly associated with *VHL* mutation.	With
Anari et al., 2022 [29]	An MRI-based radiomics model to predict clear cell renal cell carcinoma growth rate classes in patients with VHL syndrome	73	MR	*VHL*	They implemented a machine learning algorithm utilizing the radiomic features of renal tumors identified on baseline MRI in VHL patients to predict the volumetric growth rate category of these tumors. They have demonstrated that machine learning employing radiomic features from a baseline MRI can be utilized for accurately predicting tumor growth rate (volumetric doubling time) in VHL patients (mean AUC-ROC of 0.795). The accuracy for predicting tumor doubling time classes was not different among the MRI sequences (*p* = 0.56).	With
Greco et al., 2022 [30]	Clinicopathological and Body Composition Analysis of *VHL* and *TTN* Gene Mutations in Clear Cell Renal Cell Carcinoma: An Exploratory Study	54	CT	*VHL*, *TTN*	Gene expression and overall survival was assessed on a large cohort of 483 patients and 533 tumor samples. There was a statistically significant difference in the *VHL* expression due to the decrease in *VHL* expression in pathological specimens compared to normal renal tissue. ccRCC patients with a *VHL* low/medium expression show a survival probability greater than 0.25 and a maximum survival greater than eight years. Moreover, the results showed a significant decrease in TAT, VAT, SAT, and TAM in the ccRCC-*TTN* group compared to the ccRCC-*VHL* group. A statistically significant difference of the *VHL* expression reduction in primary tumor (*p* < 0.0001) and a *TTN* expression increase in primary tumor (*p* < 0.0001) was shown. Statistically significant differences between the two groups were obtained for TAT (*p* < 0.01), VAT (*p* < 0.05), SAT (< *p* < 0.05), and TAM (*p* < 0.05) areas.	Without

Abbreviations: AUC, area under curve; *BAP1*, BRCA-1 Associated Protein-1; ccRCC; clear cell renal cell carcinoma; CT, computed tomography; *FBN2*, fibrillin-2; FDR, false discovery rate; FLG, filaggrin; *KDM5C*, lysine-specific demethylase 5C; MCMO, multi-classifier multi-objective; MR, magnetic resonance; MRI, magnetic resonance imaging; mRMRe, minimum redundancy maximum relevance ensemble; *MUC4*, mucin-4; *MUC16*, mucin-16; OS, overall survival; *PBRM1*, protein polybromo-1; RCC, renal cell carcinoma; RF, random forest; ROC, receiver operating characteristic curve; SAT, subcutaneous adipose tissue; *SETD2*, SET domain containing 2; TAM, total abdominal muscle; TAT, total adipose tissue; TCGA-RCC, The Cancer Genome Atlas-renal cell carcinoma; TCGA-KIRC, The Cancer Genome Atlas-kidney renal clear cell carcinoma; *TTN*, titin; VAT, visceral adipose tissue; *VHL*, von Hippel–Lindau.

## Data Availability

Not applicable.
